# Robot education peers in a situated primary school study: Personalisation promotes child learning

**DOI:** 10.1371/journal.pone.0178126

**Published:** 2017-05-23

**Authors:** Paul Baxter, Emily Ashurst, Robin Read, James Kennedy, Tony Belpaeme

**Affiliations:** 1 Lincoln Centre for Autonomous Systems, School of Computer Science, University of Lincoln, Lincoln, United Kingdom; 2 Centre for Robotics and Neural Systems, The Cognition Institute, Plymouth University, Plymouth, United Kingdom; 3 IDLab – imec, University of Ghent, Ghent, Belgium; CITEC, Bielefeld University, GERMANY

## Abstract

The benefit of social robots to support child learning in an educational context over an extended period of time is evaluated. Specifically, the effect of personalisation and adaptation of robot social behaviour is assessed. Two autonomous robots were embedded within two matched classrooms of a primary school for a continuous two week period without experimenter supervision to act as learning companions for the children for familiar and novel subjects. Results suggest that while children in both personalised and non-personalised conditions learned, there was increased child learning of a novel subject exhibited when interacting with a robot that personalised its behaviours, with indications that this benefit extended to other class-based performance. Additional evidence was obtained suggesting that there is increased acceptance of the personalised robot peer over a non-personalised version. These results provide the first evidence in support of peer-robot behavioural personalisation having a positive influence on learning when embedded in a learning environment for an extended period of time.

## Introduction

Social robots have the potential to make positive contributions to a range of human-centred activities, from support of the elderly to therapeutic assistance to adults and children [1–4]. One domain of particular interest is education, where social robots may be used to supplement existing teaching structures to provide additional support to children. A range of evidence comes together to support this perspective: it is known that one-to-one tutoring leads to significant learning improvements [5], classroom engagement is a predictor for peer acceptance in later years in young children [6], and that personalised social and academic support has been shown to reinforce later achievement [7]. The role of robots to facilitate engagement in classroom activities thus has potentially significant consequences for learning as well as for social development. In these efforts, the role of adaptivity is considered central to the efficacy of application: an adaptive robot will be able to take into account the specific needs, requirements and preferences of the person(s) with whom they are interacting. This personalisation of robot behaviours is the focus of the present work. In this paper, we demonstrate the positive role that personalised robot peer behaviours play (along a number of dimensions) for child learning in a situated context.

Existing work has shown that the presence of robots confers a number of advantages over other media (e.g. standard desktop computers or paper-based systems) for learning and behavioural change in people [8]. This has been demonstrated, for example, in the domains of adherence to weight-loss programmes [9], reducing puzzle solving times [10], learning words [11], and motor task learning [12]. Further studies have shown that physical robots will attract more attention than their virtual analogues [13–15], and will comply with their requests [16], following evidence suggesting children regard social robots as psychological agents [17] and are perceived as more enjoyable interaction partners [18, 19]. Taken together, these studies indicate that robots take advantage of, and amplify, the human propensity to anthropomorphise inanimate objects, which results in subsequent behavioural change [20, 21]. Given this effect of physical robots as a basis, the question of interest is therefore how the behaviour of the robot can augment this to maximise the desired outcome for the human interactant.

Two prior studies in the domain of social robots for educational contexts have set benchmarks for subsequent research. In the first (single experimental condition) study, a robot was placed in a corridor outside two Japanese classrooms for two weeks (6-7 and 10-11 year-olds, under experimenter supervision), with the nominal task of encouraging the children to learn English in unstructured interactions in break times [22]. This study demonstrated significantly increased vocabulary recall by the children. In the second study, a humanoid robot with a gradually unfolding repertoire of social behaviours was placed within a classroom of 10-11 year-olds in Japan for two months (32 experimental days), although interactions took place outside of normal lesson times and also under constant experimenter supervision [23]. While the examination of learning outcomes for the children was not the focus of the study, with the development of relationships between the children and robot the primary aim, it was shown that children who maintained peer-like interactions with the robot maintained interactions over the extensive experimental period. Extending significantly from these works, the present study focusses explicitly on learning, and being simultaneously embedded both physically and in terms of the curriculum in the classroom itself.

A number of other studies have recently followed from these seminal works to further explore the specific potential role that such social robots can play in helping *children* to *learn*, although typically these have taken place outside of school classrooms or over isolated interaction sessions. While a number of studies demonstrate the benefit of social robots in terms of preference [24] and for adult learning [25], studies with children have shown that personalisation of robot behaviour (e.g. using names) [26] and task content (e.g. increased coverage of subjects in which the children struggle) [27] can lead to modest learning gains in short-term and single interactions, and that collaborative learning between children is facilitated [28]. However, these studies are ambiguous regarding the actual impact of social behaviour on child learning: the presence of robots appears to facilitate increased learning, but the role of social behaviour to extend this effect remains unclear, in contrast to the human-centred theory [29].

In the present work, we specifically examine the role that robot personalisation can play in supporting the learning of children in social interaction with a humanoid robot over longer and more intensive periods of time. We conduct this study within the classrooms themselves, integrated within the school curriculum, and with no experimenters present during proceedings, so as to maximise the ecological validity of our observations, results, and potential utility for real applications. Our findings broadly support the hypothesis that personalisation within interactions facilitates learning.

## Situated school study

In an education context, robots could take on a number of social roles, such as tutor or peer, each of which gives rise to certain behavioural expectations. As noted above, both have been found to result in child learning, and both come with the expectation of social behaviour [30, 31]. However, whereas a tutor can be reasonably expected to not make mistakes, there is not necessarily such an expectation for a peer: indeed, it has been found that the robot making mistakes will further encourage child learning [32]. A robot with a more cooperative interaction style has been found to elicit higher levels of engagement when interacting with children [33]. Finally, in terms of preferences, it has been shown that in comparison with a tutor, a peer role is preferred [34]: in the domain of robot companions for diabetic children for example, the robot playing the role of a peer appears to be preferred over a tutor [4, 35]. For the present study, we therefore focus on the role of social robot as peer; a learning companion.

This focus on the peer role entails a greater emphasis on collaborative (involving multiple parties attempt to learn something together [36]) rather than didactic (in the manner of a teacher) interactions between the child and robot. Technology is broadly being highlighted as a means of ameliorating this [37]: child-child interaction studies have shown that collaborations are more effective with jointly visual and manipulable objects [38]. The touchscreen-based task environment we use takes advantage of this effect by implicitly constraining the content of the interaction to the task [39], thus encouraging collaboration and participation (active learning) [40] in a shared task space. It has been previously shown how such a task environment provides an engaging context for child-robot interactions [15, 41, 42].

Our application context is a primary school classroom, with the intent that the robots act autonomously whilst embedded within them. We seek to achieve ecological validity for the study [43]: we emphasise that the robots are not under experimenter supervision during the experiment (the teacher themselves provide this) and thus also not whilst the children interact with the robot, as this detracts from relevance to potential deployment scenarios. Furthermore, we consider the robot to be embedded within the classroom, both in terms of physical presence (in the classroom, and in operation during lesson time), but also in terms of the incorporation of learning material from the children’s curriculum. These two points (embeddedness and unsupervised operation) constitute novel extensions to studies in the existing literature.

These considerations contextualise the broad hypothesis of the present study: *that personalisation in a robot learning peer will lead to greater learning effects for children in an embedded educational context*. Four aspects of this broad hypothesis require specification. Firstly, we hold learning to incorporate generalisation in addition to memorisation, following a revision of Bloom’s taxonomy [44], which identifies cognitive processes (from remembering to creation) as well as knowledge (from factual to meta-cognitive) as essential educational objectives. Our learning evaluation thus specifically incorporates aspects of application of knowledge to a new context. Secondly, we note that there are a range of potential targets for learning for the children in their educational environment. For this reason, we examine both topics that are part of their existing curriculum (familiar subjects), and ones that are not (novel subjects). Thirdly, the novelty of our classroom-embedded application necessitates an examination of the attitudes of the children in addition to their performance, to begin to assess the wider implications of such an application. We thus attempt to characterise the wider experience of the children over the experimental period. The fourth aspect is the nature and extent of robot behaviour personalisation, which has been stated as “…reflect[ing] the needs and requirements of the (social) environment where the robot is operating in” [45] (p20). Consistent with this definition, Lee et al [24] describe three non-exclusive means of increasing robot personalisation that include aspects of behaviour that are not related directly to adaptation per se, but also to the creation of a *personable* character: increasing friendliness, alteration to fit user preferences, and adaptation over repeated encounters. This indicates a broad and integrated perspective on personalisation; a position that we here subscribe to.

A range of evidence in HRI studies, grounded in multiple other disciplines, may be brought together to further support this perspective for the present work. Mapping onto the definition and characterisation of behaviour personalisation discussed in the previous paragraph, we identify three particular facets of personalisation that are particularly relevant to our task context: adaptation of non-verbal behaviour, personable language content, and alignment to task performance. These encompass both adaptive (non-verbal and task performance adaptation) and personable (language content) behaviours that match the social interaction context (repeated peer-peer interactions in an education setting). Following the phenomenon that humans align their actions to one another, such as linguistic content [46], non-verbal behaviour adaptation follows from and encompasses those aspects of the robot behaviour that are manipulable based on observation of the child’s behaviour [47], based on the phenomenon that humans will adapt their behaviour to that of a robot [48]. Personable language content refers to the explicit taking into account of the specific person with which the interaction takes place: for the present study, this entails using the interacting child’s name during the interaction [26], and using an informal style for instruction and feedback utterances [49]; being personable as opposed to imperative. Finally, performance alignment is the modification of aspects of the task to align them with the performance of the child [25, 50]. In the present study, such performance alignment is employed at two levels: firstly at the task level, where the children could repeat an individual task, and secondly at a behavioural level, where the performance of the robot is aligned with that of the child [47]. The first and third facets of personalisation effectively constitute a memory of prior interactions, which may subsequently be applied to further interactions.

As stated above, we consider these three facets of personalisation together as a single concept [24]. Evidence from a range of sources indicates that the consideration of single modality interaction cues is insufficient to account for human behaviour, and that instead a fundamentally integrated perspective needs to be taken [51]. For example, emotion perception has been found to require conceptual processing, and is thus open to contextual influences (e.g. visual and social) [52]. Furthermore, recent theoretical developments in the domain of social cognition, emphasising contingent behaviours, suggest that the context of the interaction shapes the individual’s disposition to engage in interaction, resulting in a difficulty in handling out-of-context cues [53]. Given that the context is at least partly determined by the interaction partner, this further indicates the importance of coherency of context. Human social interactions naturally integrate all these aspects of personalisation, and so we anticipate that such coherency would also be expected of a nominally social robot. Taken together, and as a first truly embedded study of this type, these lines of evidence motivate and justify our decision to maintain the integration of the three facets of personalisation for the present study.

**Fig 1:**
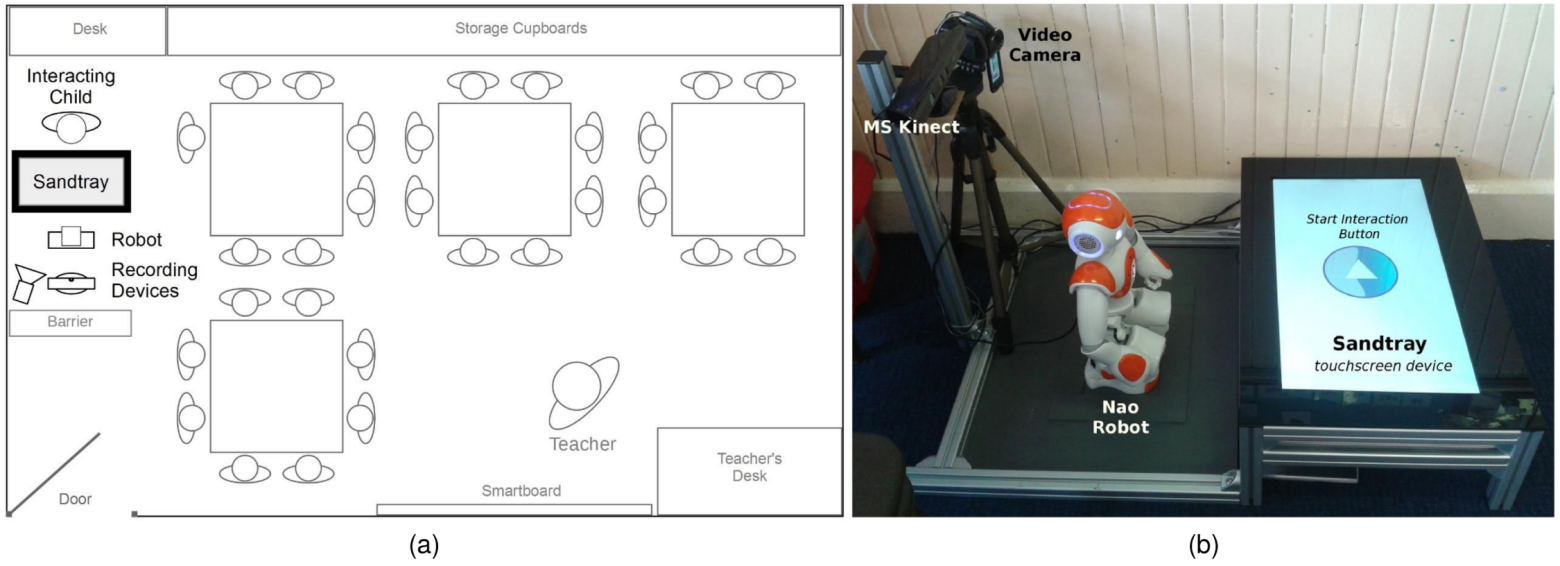
Typical physical setup of the system within the classroom. The robot, Sandtray—a touchscreen device—and camera setup was located in one corner of the room in which the children had their normal lessons. Interactions took place during normal lesson time. Both classrooms had similar arrangements. *Not to scale.*

The study described in this paper seeks to address the broad hypothesis by using a two-condition, between-subject experimental design. Two age- and ability-matched groups of 7-8 year-old children in a U.K. primary school form the subject groups. A single robot is deployed in each group in the same room in which the children engage in their daily lessons (Fig 1), during which time individual children interact with the robot. They engage in a collaborative sorting task with the robot on novel (history—the stone age) and familiar (mathematics—times-tables) topics using a large mediating touchscreen [54] (Fig 1(b)). There are no experimenters present during the interactions, which took place over a continuous two-week period. In the “*Personalised*” condition (**P**), the robot personalises its behaviour along the three defined dimensions; in the “*Non Personalised*” condition (**NP**) the robot displays non-adaptive, non-personalised behaviour (see the Methods section for details).

## Materials and methods

The aim of the study conducted was to investigate whether personalised robots embedded within a classroom for an extended period of time (part of normal classroom activities, and with no experimenters present) can lead to increased child learning. The primary hypothesis of the study is therefore that children in the Personalised robot condition would learn more than children in the Non-Personalised robot condition, on the given set of topics. In addition to this, we seek to explore some of the wider implications of having the robots embedded within the classrooms, and whether the personalisation had any additional effects beyond the target learning outcomes.

### Ethics statement

Approval for conducting this study was granted by the Plymouth University Faculty of Science and Technology Human Ethics Committee, as part of a thematic programme of research involving the robot and touchscreen setup, and children in local schools. An opt-out informed consent was obtained in writing from the parents/guardians of all participating children, and a separate opt-in written informed consent was obtained for video recording the interactions between the children and the robots. Children were withdrawn from the study if consent was not obtained, and it was made clear that they could withdraw if and when they wished to.

### Subjects

A total of 59 children aged 7-8 (in U.K. year 3) took part in the study (summer term). All children attended a single U.K. primary school, but were divided into two classes. This division was not on the basis of ability. Gender balance favoured girls, although this applied equally to both the first (12 boys, 18 girls, 30 in total) and second (12 boys, 17 girls, 29 in total) classes.

Each class was based in a different room where the majority of their lessons took place (Information Technology lessons and Sports took place in different areas of the school). These classrooms were located on the same corridor on the first floor of the school building (one other empty classroom was on the same floor). The children in the two classes were separated in these classes, although break times were held in communal areas of the school. Each class was randomly assigned an experimental condition for the duration of the experiment. Each class had a separate teacher who remained with the class for the duration of the experiment period. In addition, each class was assigned a teaching assistant (TA), who varied by day. Both teachers and TAs were briefed regarding the experimental setup; none of these were told of the experimental conditions, nor that there were different robot behaviours deployed in the two classes. This arrangement of children and classes provided the greatest degree of homogeneity possible between the conditions by controlling for a number of potentially confounding subject and environment factors.

### Materials

The same hardware setup was employed in both classrooms (Fig 1(a)). This consisted of a touchscreen (the Sandtray), Nao humanoid robot (58cm tall, made by Aldebaran Robotics), aluminium extrusion frame, and recording devices (Fig 1(b)). The robot and touchscreen were synchronised over a wireless network such that the robot could manipulate virtual ‘objects’ displayed on the screen [54]. The aluminium frame served the dual purpose of maintaining the arrangement of the equipment (e.g. reducing cable trip hazards) and providing a minimal barrier to discourage the children from interfering with the hardware. The only difference between the robots used was the highlight colour of the plastic panels: orange was used in the Personalised condition, and grey was used in the Non-Personalised condition. One such hardware setup was deployed in each classroom, where it remained for the continuous two week period of the experiment.

### Learning task

Taking into account the children’s current curriculum, two topics for learning in the interaction with the robot were chosen, since there is a suggestion that multiple activities support the maintenance of engagement [55]. The first was *novel* to the children, but was due to be learned in the following academic year. The second was *familiar* as it had already been the ongoing subject of learning. This dual-topic learning task was chosen to assess whether, in the context of a familiar learning environment, a robot learning companion could be applied as an intervention for an existing learning process as well as to a novel task.

The familiar learning task was chosen to be the times-tables, up to and including 12. This formed part of the curriculum that the children studied throughout the year. As such, the children were used to the concept involved, but varied in ability across the subject group. The novel learning task concerned the stone age. This was a new subject matter for the children in the school environment, with it due to appear on the syllabus in the following year. Learning gains made in this topic would thus have been beneficial to the children in the future.

**Fig 2:**
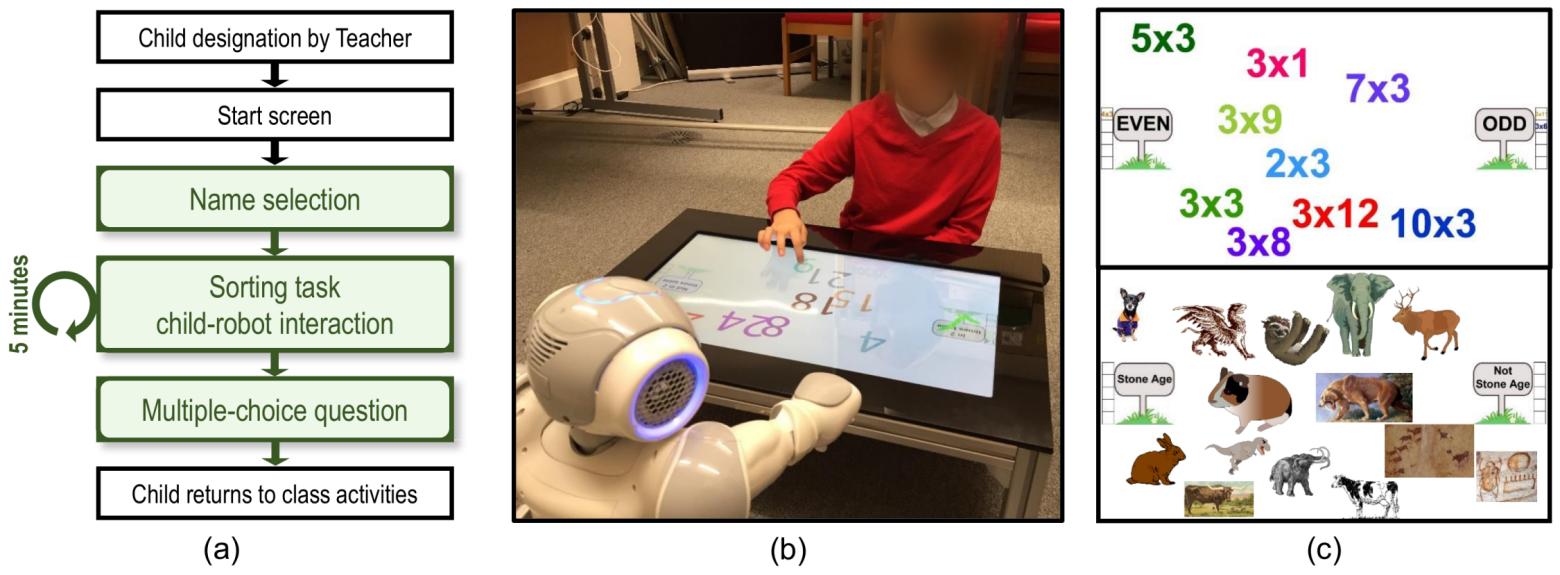
Interaction structure and contents. (a) structure of each interaction, with five minutes on the collaborative sorting task itself; (b) example of a child engaged in the task with the robot (hardware and classroom setup as shown in Fig 1); (c) two sample image libraries, showing a 3 times-table task, and a stone-age animals task.

Both topics were administered using the Sandtray, and were structured in the form of a series of two-category sorting tasks played with the robot (e.g. Fig 2(c)). A library of images is placed on the screen, each library comprised of two static category images, and a number of movable images. The task is to sort each movable image into the correct category: visual feedback is displayed on the screen to indicate a correct (or incorrect) categorisation. The child uses the touchscreen, and the robot can virtually drag the same images, thus establishing parity of potential interaction affordances with the screen, and facilitating interaction between the child and robot [54]. This methodology has been employed in a number of previous studies [4, 15] and has proven to be an effective strategy to engage children with robot interaction tasks. Given that both novel and familiar learning tasks are displayed on the touchscreen, the tasks are interleaved: i.e. times-tables and stone age libraries are alternated (Table 1).

**Table 1:** Image libraries used for the sorting tasks. Shown are the type of sorting task for each library, and the categories used for the sorting itself. There were 14 images per stone age library, and 12 images per times-table library. *Stone age libraries are in italics: the fifth and sixth of these were combinations of images from the first four stone-age libraries*.

Library	Library topic	Library contents	Sorting task
1	Times-table	2x table	In/Out
*2*	*Stone age*	*Lifestyle*	*Yes/No*
3	Times-table	10x table	In/Out
*4*	*Stone age*	*Animals*	*Yes/No*
5	Times-table	5x table	Odd/Even
*6*	*Stone age*	*Tools*	*Yes/No*
7	Times-table	2, 10 & 5 division	Odd/Even
*8*	*Stone age*	*Art*	*Yes/No*
9	Times-table	3x table	Odd/Even
10	Times-table	4x table	In/Out
11	Times-table	6x table	In/Out
12	Times-table	3, 4, & 6 division	Odd/Even
*13*	*Stone age*	*mix of subjects*	*Yes/No*
14	Times-table	7x table	In/Out
15	Times-table	8x table	In/Out
16	Times-table	9x table	Odd/Even
17	Times-table	11x & 12x tables	Odd/Even
*18*	*Stone age*	*mix of subjects*	*Yes/No*

The image libraries were the same for all children, in both conditions. Each image library formed a two-category sorting task, of which half were uniquely associated with one of the two categories, and half to the other. The stone-age libraries were each comprised of 14 images, and the times-tables libraries were comprised of 12 images. The images appearing in the image libraries did not appear in the pre- and post-experiment knowledge tests. The order of the times-tables is according to difficulty (as specified by the teachers prior to the study), whereas the stone-age image libraries each covers a different topic (where the task is to recognise whether each image displayed belongs in the stone-age or not).

There were two additional learning-related components that were tested in this experiment. In the first, an item of factual information was stated by the robot to the children during their interaction, with recall of this fact tested for at the end of the experiment (with the multiple-choice question “how long ago was the stone-age?”, options: {two years, two hundred, two thousand, two million, two trillion, two bazillion}; last option a fake large number, correct answer is two million years ago). The second component was tracking child performance in a class-based task that was independent of either the familiar or novel learning tasks (*incidental* task): spelling test scores were chosen as they were assessed on a weekly basis. In this way, performance prior to, during and after the experiment could be tracked.

### Conditions

Two experimental conditions were employed: a Personalised (P) interactive robot condition, and a Non-Personalised (NP) robot condition. The robot behaviour differed between the robots in three distinct respects: non-verbal behaviour (gaze, movement alignment), verbal behaviour (friendliness, personalisation), and adaptivity of progression through the learning content (to personal performance). In neither condition were the children or teachers made aware of the differing aspects of behaviour, nor of the differences between the conditions. In both conditions, the robots acted autonomously, i.e. not under the control of an experimenter or teacher.

In the Personalised condition, the robot was animated (actively seeking to match gazes to it by the interacting child, and exhibiting life-like idling movements), responsive to the approach of a child at the start of an interaction (it would stand up), and varied its behaviour according to the characteristics of each child, as observed in the interaction. In terms of non-verbal behaviour, this constituted adaptation of the drag speed of the robot movements on the screen, the accuracy of the movements (in terms of percentage correct and incorrect categorisations), and the length of time between successive moves [47]. In terms of verbal behaviour, the robot would use the interacting child’s name, and employ a more friendly (as opposed to imperative) demeanour. Full details may be found in the supplementary materials (S1 File). Progression through the lesson image libraries was partially dependant on performance: assuming that the child completed more than four image categorisations, then the image library was considered to be successfully completed if the success rate for the child (i.e. not including robot moves) exceeded 65%, with performance below this resulting in the library being repeated (up to a maximum of three times). This personalisation of lesson progress provides a greater degree of opportunity for practice on those topics where performance was low.

For the Non-Personalised condition, the robot’s behaviour remained constant throughout all interactions, independent of the characteristics of each child, and was not responsive to the approach of a child. This included movement speed, accuracy of moves, and delay between moves. Imperative non-personal phrases were used (matched for number and length of utterances used in the Personalised condition), and the progression through the learning material was set at a constant rate for each child: each image library was completed only once before moving on.

In neither condition was there a mechanism to explicitly consider turn-taking behaviours; nevertheless, previous work has indicated that if the children perceive the robot to be a social agent, turn-taking will emerge in the interaction [41].

### Protocol

The class teachers were not informed of the hypotheses of the study, nor of the differences in robot behaviour between the classrooms. The teachers administered pre-experiment knowledge tests and questionnaires, and did so again for post-experiment tests and questionnaires. During the experiment period itself, the teachers collected child performance on the normal spelling tests and maths times-table tests, which were administered weekly. Maths lessons were postponed for the two-week duration of the experimental period. A final debriefing interview was conducted with the teachers after the experimental period. These additional data were collected to enable a broader perspective on the influence of the robot in the classroom beyond the interactions themselves.

During the experiment, there were no experimenters in the room: the robot system ran autonomously, with experimenters only present at the start and end of the day to initialise and shut down the system, respectively. In both conditions, the teachers designated the next child to interact with the robot. The child would approach the robot setup (from the right-hand side of Fig 1(b) for example), kneel down, and press a large ‘start’ button on the screen. Following a verbal acknowledgement from the robot (differing by condition), the child would then proceed to select their name on the screen. On name confirmation, the robot would begin the interaction (differing by condition) with the last uncompleted image library.

After five minutes of interaction time, during which both the child and robot were able to sort the images on the screen, the robot would announce that it had to rest (differing by condition). The child would be asked to answer a multiple-choice question on the screen, the robot would return to it’s rest position, and the child would return to their seat in the classroom. The next child could then be called to interact by the teacher.

### Metrics

Four types of metric were used: pre- and post-experiment knowledge tests, within-interaction performance data, questionnaires assessing opinion of and engagement with the robot, and measures of performance in the classroom not involved in the experiment.

The pre- and post-experiment knowledge tests were administered on paper on the subject of the novel learning task. They consisted of 24 images, 12 of which belonged to the stone-age category, 12 did not. The same test was administered for both pre and post, but the children were not given any feedback after the pre-test; the images in the test did not appear in the robot interaction stage (Table 1), thereby testing an aspect of generalisation.

Within the interactions, all aspects of the child’s performance as detectable by the touchscreen and robot were logged. This included the number of correct and incorrect classification attempts per image library (including repeats in the Personalised condition). The change in performance over interaction time per child could therefore be assessed. In addition to this, at the end of each interaction, the child was asked to answer a multiple-choice question on the screen before returning to their seat in the classroom (Table 2, the precise phrasing depended on the condition, shown in table S1 File). The questions after interactions two and three were same in order to explore the changes in response over time. The questions varied according to the interaction number, and are shown in Table 2. If the child did not respond within 30 seconds, the interaction would end, and a ‘no response’ entry was made.

**Table 2:** End-of-interaction questions. Multiple choice questions displayed on the screen after each interaction, each of which had five possible responses.

Int.	Question	Option 1	Option 2	Option 3	Option 4	Option 5
1	Did you enjoy playing?	Not at all	No	A bit	Yes	Yes a lot
2	What would you prefer to play with next?	Robot	Classmates	Read a book	Play outside	Games console
3	What would you prefer to play with next?	Robot	Classmates	Read a book	Play outside	Games console
4+	What do you think of the robot?	Boring	OK	Good	Bad	Brilliant

The third type of metric used was the administering of standard questionnaires. A preliminary pre-study questionnaire was administered to provide an indication of prior expectations, following prior work [23]. The main battery of questionnaires was administered after the experiment had been completed. Three questionnaires were used at this time. The first was comprised of two sub-scales of the Intrinsic Motivation Inventory [56, 57]: interest/enjoyment and perceived competence. The second was to assess the perception of social presence of the robot [58], as previously validated [59]. The third was to assess the perceived social support provided by the robot [60], an adaptation of a version validated with children (peer subscale) [61]. All questionnaires may be found in the supplementary materials (S1 File).

The final evaluation metric was performance of the children in a classroom task not related to the topics of the familiar and novel learning tasks. Spelling was determined as a suitable choice for this as it was assessed on a weekly basis, which allowed change in performance to be tracked over the course of the experiment.

### Data analysis

For all results, the 95% confidence interval (CI) is provided for both within condition data and between condition comparisons. Where appropriate, normality of data is tested for using the Shapiro-Wilk test [62]; unless otherwise stated, the data are found to be consistent with normality, if not, then the Wilcoxon (non-parametric) test was employed. Homogeneity of data variance is tested for using the Levene’s test [63]. Bootstrapping is employed to provide estimations of population hypothesis testing from our collected sample [64]: 10^6^ replications are used and the studentized bootstrap 95% CI reported [65].

When considering learning effects, it should be noted that the pre- and post-tests used have a maximum (and minimum) possible score, leading to a negative correlation of absolute learning gain and pre-test score [66]. Given this limit on maximal attainable increase in score, the normalised learning gain metric, *g* = (*score*_*post*_ − *score*_*pre*_)/(*score*_*max*_ − *score*_*pre*_), is employed, which normalises change in score to pre-test score, while being uncorrelated with pre-test score [67]. This enables an assessment of the extent of learning irrespective of prior (starting) performance. Normalised learning gain is calculated for all individuals, with the mean normalised learning gain for each condition subsequently derived (and associated 95% CI).

## Results

Two primary aspects of the results are considered. Given the main hypothesis, the effect of the personalisation of robot behaviours on learning outcomes is considered. Then, given the continued presence of the robots in the two classrooms for the two week period, an assessment is made of how the children’s perceptions varied over time, both within and between conditions. All data may be found in the supplmentary materials (S2 File). First however, we summarise the characteristics of the interactions in the two conditions.

### Expectations and interaction characteristics

As part of the pre-experiment questionnaires, the expectations of the children were assessed, following [23]. Four questions were asked of the children regarding their perceptions of the robot and how they expected their interactions to be (please refer to S2 File for full wordings and possible responses). The results of this show no effective differences between the two conditions, reinforcing the notion that the subject population is equivalent between conditions. The children generally expected the robot to be like a friend (66.7%, followed by games console, 15.8%, and toy, 10.5%), wanted to know how the robot worked (across conditions, scale 1–5, *M* = 4.53, *n* = 59, 95% CI = [4.34, 4.72]), and wanted to be friends with the robot (across conditions, scale 1–5, *M* = 4.71, *n* = 59, 95% CI = [4.57, 4.85]).

Both robot setups were permanently located in the two classrooms for a two week period. This encompassed nine school days (a school closure occured on one day in the second week). Over the two conditions for the experimental period, a total of 199 interactions took place between the children and the robots—note that each of these took place in the classroom during normal lesson time, and thus other children were present (albeit under the direct supervision of the teacher). Overall, the children completed an average of *M* = 4.56 image libraries (*n* = 59, *SD* = 1.10) per interaction with the robot.

**Fig 3:**
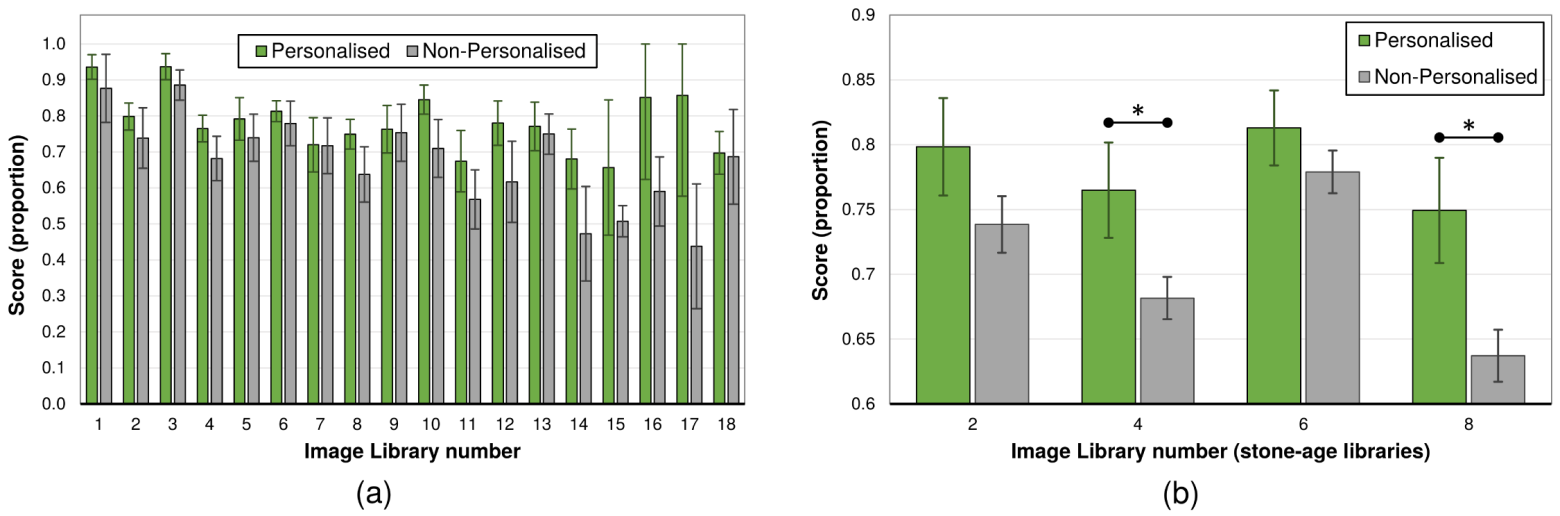
Library scores per image library. Overview of mean scores per library, by condition, error bars are 95% CI: (a) performance in each of the image libraries, see Table 1 for library contents; (b) scores for the first four stone-age image libraries (novel subject): ‘*’ denotes significance at the .05 level.

Given the touchscreen-centred nature of the interactions, performance of the individual children on individual image libraries could be recorded and compared between conditions. This progression through the image libraries is shown in Fig 3. In all cases, performance in the Personalised condition exceeds that in the Non-Personalised condition, however, significance is only present in a few of these cases (S2 File). While not a statistically significant effect, note that the difference between the conditions generally increases as progression through the image libraries increase.

### Learning outcomes

Three learning topics were considered, and one recall task. The *novel* topic was recognition of stone-age items; the *familiar* topic was the maths times tables (from two to twelve, inclusive); and the *incidental* topic was a weekly spelling test. The recall task was a fact introduced by the robot in its interactions with the children, the memory for which was tested after the experimental period.

The two classes used in this study were not divided on the basis of ability, although they were of the same age. In order to verify that the abilities of the children involved were ability matched with respect to the learning metrics used, we consider the pre-experiment scores in each of the three topics examined. Each of these indicates that the performance is indeed similar in the novel (*M*_*P*_ = 0.731, *n*_*P*_ = 30, 95% CI = [0.695, 0.766], *M*_*NP*_ = 0.759, *n*_*NP*_ = 29, 95% CI = [0.718, 0.799], independent samples two-tailed t-test: *t*(57) = 1.097, *p* = .277), familiar (*M*_*P*_ = 0.557, *n*_*P*_ = 30, 95% CI = [0.478, 0.635], *M*_*NP*_ = 0.520, *n*_*NP*_ = 29, 95% CI = [0.467, 0.574], independent samples two-tailed t-test: *t*(57) = 0.821, *p* = .415) and incidental tasks (*M*_*P*_ = 0.617, *n*_*P*_ = 29, 95% CI = [0.526, 0.708], *M*_*NP*_ = 0.654, *n*_*NP*_ = 28, 95% CI = [0.553, 0.755], independent samples two-tailed t-test: *t*(56) = 0.437, *p* = .664). This justifies the examination of differential learning outcomes in the two conditions.

**Fig 4:**
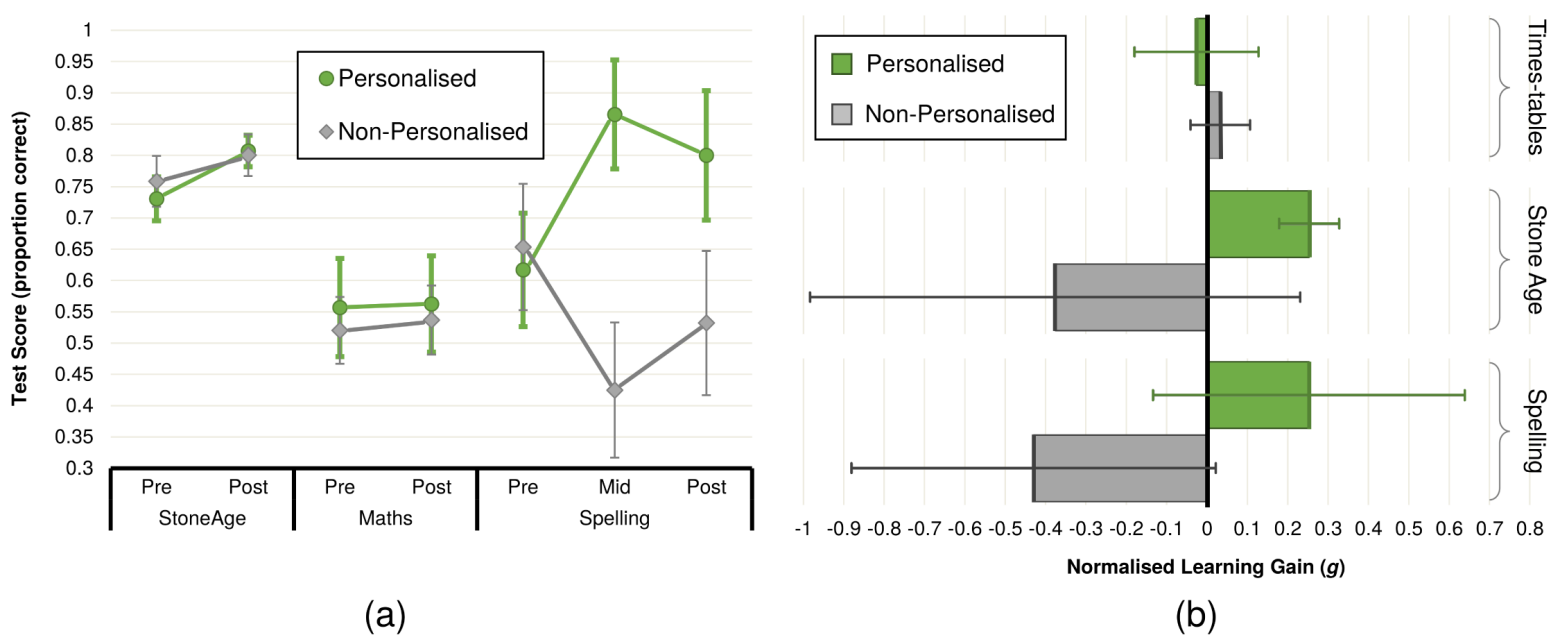
Child learning performance between conditions. (a) summary of mean percentage test scores (for pre and post experimental period) for the familiar learning task (times-tables), the novel learning task (the stone age), and the independent task (spelling, for which there was also a mid-experiment test); (b) normalised learning gain exhibited in the familiar, the novel, and the independent learning tasks. Error bars show 95% CI.

From the pre-test scores described above, consideration of the post-test scores provides an initial and illustrative indication of the change in performance. For the novel (*M*_*P*_ = 0.807, *n*_*P*_ = 30, 95% CI = [0.782, 0.832], *M*_*NP*_ = 0.800, *n*_*NP*_ = 24, 95% CI = [0.767, 0.834]), familiar (*M*_*P*_ = 0.563, *n*_*P*_ = 30, 95% CI = [0.485, 0.640], *M*_*NP*_ = 0.537, *n*_*NP*_ = 27, 95% CI = [0.481, 0.592]) and incidental (*M*_*P*_ = 0.800, *n*_*P*_ = 29, 95% CI = [0.697, 0.903], *M*_*NP*_ = 0.532, *n*_*NP*_ = 28, 95% CI = [0.417, 0.648]) tasks, this indicates similar outcomes between conditions (Fig 4(a)). Only in the incidental task is there an indication of a significant difference between the conditions in the post-test (independent samples two-tailed t-test: *t*(55) = 3.396, *p* = .0013).

However, consideration of only the difference between pre- and post-test scores (whether by group or by individual) is a flawed metric since there is a ceiling on the maximum attainable score (100%), and thus also on the maximum attainable increase in score given a pre-test score. To counter this issue, we employ the ‘normalised learning gain’ metric (see Methods section), which normalises score change to pre-test score. Applied to all subjects in both conditions (i.e. all children in the study, minus exclusions), this indicates no significant learning results for the novel (*M* = −0.026, *n* = 54, 95% CI = [−0.309, 0.256]), familiar (*M* = 0.002, *n* = 57, 95% CI = [−0.085, 0.090]) or incidental (*M* = −0.082, *n* = 59, 95% CI = [−0.379, 0.214]) learning tasks.

Applied on a condition-basis (Fig 4(b)) to the data shows that for the novel task (stone-age) the 95% confidence interval around the observed mean learning gain for the Personalised condition does not include zero (*M*_*P*_ = 0.253, *n*_*P*_ = 30, 95% CI = [0.179, 0.328]), whereas the Non-Personalised condition does (*M*_*NP*_ = −0.376, *n*_*NP*_ = 24, 95% CI = [−0.983, 0.231]). For the familiar (*M*_*P*_ = −0.026, *n*_*P*_ = 30, 95% CI = [−0.179, 0.128], *M*_*NP*_ = 0.033, *n*_*NP*_ = 27, 95% CI = [−0.040, 0.107]) and incidental (*M*_*P*_ = 0.253, *n*_*P*_ = 28, 95% CI = [−0.133, 0.639], *M*_*NP*_ = 0.429, *n*_*NP*_ = 27, 95% CI = [−0.881, 0.022]) tasks, all confidence intervals include zero, indicating that no learning is not an unexpected event (i.e. no significant learning effect).

A bootstrapping process was applied to provide estimations of population hypothesis testing, examining whether the observed difference between the condition means lies outside of the non-parametric bootstrapped distribution (Table 3). The analysis shows that this is the case for the novel (*M*_*P*−*NP*_ = 0.629, 95% CI = [−0.557, 0.589]) and the incidental (*M*_*P*−*NP*_ = 0.682, 95% CI = [−0.588, 0.589]) learning tasks, indicating positive learning effects in these learning tasks. This is not observed in the familiar learning task (*M*_*P*−*NP*_ = −0.059, 95% CI = [−0.174, 0.175]).

**Table 3:** End-of-Interaction Questions Bootstrapping. 10^6^ replications on the difference between the conditions (P—NP), compared to observed difference. Numbers in bold denote that observed difference of means lies outside of the bootstrapped 95% CI of the difference of means.

Metric	Difference of the Mean *(P—NP)*	95% CI of bootstrapped difference of means
StoneAge Learning Gain (*novel task*)	**0.629**	[−0.557, 0.589]
Maths Learning Gain (*familiar task*)	−0.059	[−0.174, 0.175]
Spelling Learning Gain (*incidental task*)	**0.682**	[−0.588, 0.589]
Social Presence Questionnaire	0.184	[−0.368, 0.368]
Social Support Questionnaire	0.249	[−0.395, 0.396]
IMI Interest/Enjoyment Questionnaire	0.177	[−0.328, 0.333]
IMI Perceived Competence Questionnaire	0.016	[−0.460, 0.464]

The final learning-related metric applied was a recall task. After the second image library (the first stone-age library, see Table 1), the robot would introduce a fact related to the stone-age: how long ago it was. In the experiment post-test (paper-based), a multiple-choice question (six options, see Method section) assessed retention of this fact: correct responses in the P condition (57.1%) exceed those in the NP condition (48.1%), both of which exceed chance (1/6, 16.7%). Application of the Fisher exact test (due to small/null values present in the 6x2 contingency table) reveals a marginal effect (*p* = .059). Collapsing the contingency table into 2x2 (correct/incorrect responses) reveals no significant effect (*χ*^2^(2, 55) = 0.446, *p* = .504). That both condition groups of children perform greater than chance (multinomial probability for both P and NP given 1/6 chance level, *p*<.001) indicates a learning effect. However, given the presence of the robot in the classroom during the interactions, the marginal effect between the conditions could, for example, be due to social contagion effects between individuals of the class.

These results indicate that the interaction with the personalised robot leads to a significantly increased learning outcome for the children in the novel task than with the non-personalised robot, although this is not the case for the familiar task. There is a similar suggestion of increased learning performance for the incidental task, since this was assessed at the same time and in the same way for both condition groups. However, while this result is significant, we only tentatively claim the beneficial role of the personalised robot on other aspects of classroom-based work (as with the familiar task) since there are number of factors for which there was no control put in place (e.g. potential exposure to the material to be learned in the intervening time, or social interaction effects between subjects). This result does however lend significant support to a further exploration of this issue.

### Child perceptions and correlations

After each of the interactions, a multiple-choice question was displayed on the screen, with the robot asking the children to choose one of the options prior to returning to their seat (see Table 2). The question posed after the first interaction (“*did you enjoy playing?*”) reveals high levels of agreement for both conditions: 96.7% chose “*yes a lot*” or “*yes*” in the personalised condition (*n* = 30), compared with 89.7% in the non-personalised condition (*n* = 29), with no significant difference between the two. This is not a surprising result, given the initial enthusiasm due to the novelty effect.

**Fig 5:**
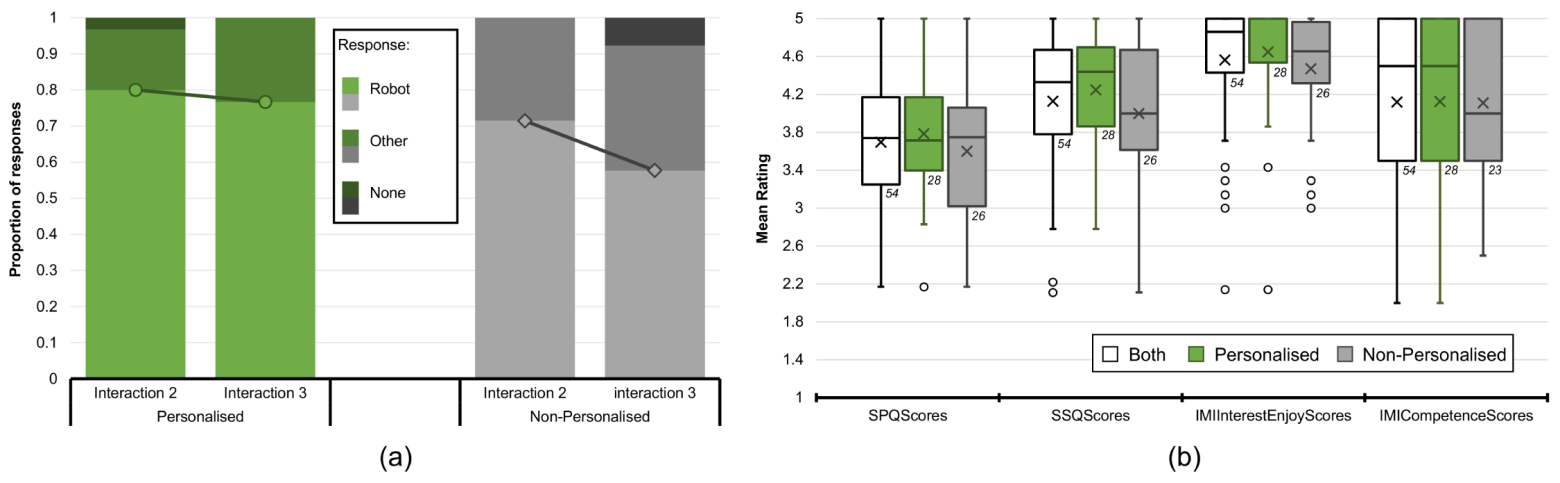
End-of-interaction question responses. (a) end of interaction responses after the second and third interactions to the question “what would you prefer to play with next?”, with “none” recorded if an answer is not given within 30 seconds (multiple choice from: robot, classmates, read a book, play outside, games console, or no answer); (b) box-plots showing child ratings for the four questionnaires (end of bars represent last datum within the 1.5*IQR; circles denote outside values; no outliers): social presence, social support, interest/enjoyment and perceived competence. Crosses indicate the mean, numbers below the bars denote sample size.

The questions posed after interactions two and three were the same (“*what would you prefer to play with next?*”, with answers classified as either robot or other), and enable an examination of changes in response over time, possibly as the novelty effect increasingly wore off. The results show (Fig 5(a)) that in both conditions there is a reduction in children choosing the robot over other options, with this effect being greater in the NP condition. This difference between interaction numbers is not significant in either the P (*d*_*int*2−*int*3_ = 0.033, *χ*^2^(2, 60) = 1.355, *p* = .508) or NP (*d*_*int*2−*int*3_ = 0.137, *χ*^2^(2, 54) = 2.703, *p* = .259) conditions. In addition, the effect size is weak for the P condition (Cramer’s *V*_*P*_ = 0.150), and moderate for the NP condition (Cramer’s *V*_*NP*_ = 0.224). These results suggest that the novelty effect was reducing over the course of the interactions.

The post-experiment questionnaires assessed four aspects of the children’s perceptions of the robot: social presence (SPQ), social support (SSQ), interest and enjoyment, and perceived competence; please refer to the supplementary materials for full details of the questionnaires (S1 File). Overall questionnaire reliability (Cronbach’s *α*) was high (listwise deletion for missing values) for the SPQ (*α* = 0.878), SSQ (*α* = 0.899), interest and enjoyment (*α* = 0.817), and for the perceived competence (*α* = 0.812), which indicates good internal consistency.

Overall, the robot was rated highly in terms of social support, the children expressed high levels of interest and enjoyment in the activity and in their own competence, with slightly lower levels of perceived social presence for the robot.

There are however no significant differences between the conditions for any of the four questionnaire-based results: SPQ (*M*_*P*_ = 3.783, *n*_*P*_ = 28, 95% CI = [3.545, 4.022], *M*_*NP*_ = 3.599, *n*_*NP*_ = 26, 95% CI = [3.311, 3.887], independent samples two-tailed t-test: *t*(50) = 0.965, *p* = .339), SSQ (*M*_*P*_ = 4.247, *n*_*P*_ = 28, 95% CI = [4.012, 4.482], *M*_*NP*_ = 3.998, *n*_*NP*_ = 26, 95% CI = [3.673, 4.323], independent samples two-tailed t-test: *t*(46) = 1.215, *p* = .231), Enjoyment/Interest (*M*_*P*_ = 4.648, *n*_*P*_ = 28, 95% CI = [4.411, 4.884], *M*_*NP*_ = 4.470, *n*_*NP*_ = 26, 95% CI = [4.239, 4.702], independent samples two-tailed t-test: *t*(52) = 1.051, *p* = .298), or Competence (*M*_*P*_ = 4.125, *n*_*P*_ = 28, 95% CI = [3.785, 4.465], *M*_*NP*_ = 4.109, *n*_*NP*_ = 23, 95% CI = [3.795, 4.423], independent samples two-tailed t-test: *t*(49) = 0.069, *p* = .945). Bootstrapping supports this by showing a lack of significant difference between the conditions with respect to these four aspects of robot perception (Table 3).

It is also of interest to examine the relationship between the performance levels, responses, and questionnaire answers. Correlations are used for this (as opposed to linear regression) since all variables are measured rather than manipulated (except for the conditions themselves): we seek to explore the data rather than generate predictions. The majority of correlations are not significant, or are the same in both conditions. However, a number of observations can be made based on the significance (or not) of the correlations in both the P (Table 4) and NP (Table 5) conditions. In the NP condition, the score attained in the first interaction is strongly and positively correlated with the first question response (whether they enjoyed the interaction: *r*(26) = 0.542, *p* = .003), whereas this is not the case for the P condition (*r*(28) = 0.097, *p* = .610), despite the mean scores (*M*_*P*_ = 0.798, *M*_*NP*_ = 0.756) and responses (*M*_*P*_ = 2.867, *M*_*NP*_ = 2.643) being equally high. Conversely, however, the response in interaction one is strongly and positively correlated with the interest/enjoyment post-experiment questionnaire response in the P condition (*r*(26) = 0.743, *p*<.001), but not in the NP condition (*r*(24) = −0.032, *p* = .877). This appears to suggest that the levels of enjoyment experienced in the first interaction are maintained throughout the experiment in the P condition, but not necessarily in the NP condition. The correlations between the post-experiment questionnaire responses are similar between the two conditions, with the exception of a significant positive correlation between perceived competence and interest/enjoyment for the P condition (*r*(26) = 0.498, *p* = .001), but not for the NP condition (*r*(21) = 0.101, *p* = .655).

**Table 4:** P-condition correlations. Pearson product-moment correlation coefficients for the P condition between the post-experiment questionnaires, first interaction score and response, and the overall learning gain. Cells in bold denote correlations significant at least at the .05 level.

	SPQ	SSQ	Int / Enj	Comp	Int1 score	Int1 resp	SA-gain	M-gain	S-gain
SPQ	1								
SSQ	**0.675**	1							
Int/Enj	**0.466**	**0.518**	1						
Comp	**0.467**	**0.378**	**0.498**	1					
Int1 score	0.094	0.042	0.026	−0.108	1				
Int1 resp	0.251	0.214	**0.743**	0.359	0.097	1			
SA-gain	0.175	−0.076	−0.208	−0.011	0.327	−0.095	1		
M-gain	−0.151	0.159	0.138	−0.234	−0.172	0.083	−0.250	1	
S-gain	−0.189	0.079	0.253	−0.102	0.127	−0.066	−0.344	0.065	1

**Table 5:** NP-condition correlations. Pearson product-moment correlation coefficients for the NP condition between the post-experiment questionnaires, first interaction score and response, and the overall learning gain. Cells in bold denote correlations significant at least at the .05 level.

	SPQ	SSQ	Int / Enj	Comp	Int1 score	Int1 resp	SA-gain	M-gain	S-gain
SPQ	1								
SSQ	**0.748**	1							
Int/Enj	**0.443**	0.335	1						
Comp	0.400	0.363	0.101	1					
Int 1 score	−0.074	0.046	−0.224	−0.307	1				
Int 1 resp	0.049	0.014	−0.032	0.142	**0.542**	1			
SA-gain	0.271	0.228	0.126	−0.001	0.079	0.207	1		
M-gain	−0.089	0.124	0.326	0.017	−0.135	−0.077	−0.157	1	
S-gain	**−0.476**	−0.311	−0.272	0.071	0.262	0.243	−0.057	0.097	1

Taken together, these results indicate a high level of continued engagement with the robot is sustained in both conditions, even after the two-week experimental period. There is some indication that, where this existed in the first place, this is sustained somewhat more in the P condition than in the NP condition.

## Discussion and conclusion

In general terms, the results show that children exhibit significantly increased learning in the novel learning task in the personalised condition compared with the non-personalised condition. This effect is also apparent in the incidental learning task, but not in the familiar learning task. Personalisation encompasses three distinct aspects (non-verbal behaviour, linguistic content, and performance alignment) that we consider as contributing to the integrated perception of a single agent: in addition to the cue integration framework [51], discontinuities between different aspects of the robot behaviour (e.g. personalisation in one respect, but not in another) may impair the overall perception [68]. This motivated our decision to provide the comparison between an integrated personalisation agent and one that did not, with the subsequently observed differences in learning outcome.

One aspect of the results that may have been impacted by this amalgamation of features in the implementation of personalisation is the perceived ‘friendliness’ of the robot, which has been characterised as including gentle, predictable movements [69]. It is thus possible that the difference in robot personalisation between conditions leads to a difference in perception of friendliness, which in turn could have an effect on the learning outcomes. However, the outcome of the post-study questionnaires indicates that that this is not the case. Specifically, the Social Presence (SPQ), Social Support (SSQ), and interest and enjoyment questionnaires all showed non-significant differences between the conditions. To the extent that the SPQ and SSQ responses are related to friendliness, this indicates that friendliness is not a confounding factor for the learning results.

In terms of behaviour, two further characteristics in particular can be incorporated beyond the three aspects of personalisation currently used, namely personality and affective responsiveness. Adaptation to personality has, with adults for example, been shown to be beneficial in the domains of the home [70], rehabilitation [71], and human-robot collaboration [72]. The incorporation of such adaptation for children in an educational context may thus be of interest in the future, even if the reliability of child self-report personality assessments may be questionable [73]. Affective responsiveness for a robot, as a more reactive phenomenon, has been associated with a greater perception of social support [60], with the face of the robot a particularly important feature [74]. A limitation in the current study regards the expressivity of the hardware platform, particularly in terms of variation in facial expression (the Nao robot used has a minimal static face, see Fig 1(b)), which limited the degree to which affective responsiveness, and hence potential for engagement [75], could be achieved. However, the present study nevertheless provides a foundation for further investigation into such issues, by establishing the importance of personalisation for learning.

The embedded nature of the present study methodology contributes to its novelty: we wish to reiterate that the robots became permanent fixtures in the two classrooms over the two week experimental period, and that there were no experimenters/technicians present with the robots during the school day. This remains a rarity in social robotics research. With only the teacher (and occasionally a teaching assistant) present with the children in the classroom, this enabled us to approximate ‘natural’ conditions for the experiment, thus supporting the ecological validity of our results. There is necessarily however a trade-off for the levels of control over potential influences in an experimental sense [43]. For example, we did not, and indeed could not given the lack of experimenter present, prevent the interaction of individual children with their classmates during their turn with the robot. Furthermore, given that the children of the two separate classes had breaks at the same time, we cannot exclude the possibility that the two groups did not exchange ideas regarding the robot and its behaviour.

The lack of significance between conditions in the familiar task may be due to four effects, apart from the possibility that there are no actual differences to be found. Firstly, robot personalisation as instantiated in the present study may not be sufficient to give rise to outcome differences, or the robot personalisation aspects used were insufficient. However, given the learning differences seen for the novel learning material, we suggest that this is not the case. We certainly acknowledge the possibility of further behavioural refinements, but the demonstration of significantly different learning gains supports our primary hypothesis. Secondly, it is possible that the novelty factor of having robots in the classroom increased overall motivation and hence performance in the tasks. This is unlikely for two reasons: (a) given the same hardware setup in both classrooms, there is nevertheless an increased performance in the novel task for the personalised condition but not the non-personalised condition, indicating the influence of condition differences over a novelty factor; and (b) the qualitative results indicate that the novelty factor decreased in the second week (also see point below). Thirdly, given the potential mixing of children between the conditions outside of the classroom as noted above, there is a possibility of some degree of cross-condition contamination. Whilst the prevalence of this is not possible to rule out, we note in mitigation that the teachers in their debriefings did not suggest that this occurred. We further note that our efforts to maximise the ecological validity of the study necessarily prevented an explicit control for the possible presence of this phenomena. Finally, we recognise that there are limitations in the administration of questionnaires to children, in terms of the ceiling effect, or social desirability distortion [76]. Although our use of standardised questionnaires mitigates the impact of this, the effect remains potentially apparent in the results (Fig 5(b)).

Nevertheless, the experimental design (developed in conjunction with the teachers themselves) sought to avoid and minimise any potential confounds. For example, the teachers were not informed of the specific hypotheses nor conditions of the study, and were involved only in the learning task content and procedural issues (to ensure that similar methods would be used by the teachers when interacting with and referring to the robot in their classroom). Similarly, the classes were balanced in terms of age, gender and ability (as evidenced by the lack of significant difference in the pre-experiment scores and attitudes), reinforced by equivalent pre-experiment expectations, resulting in homogeneous condition groups, which validates our results and observations [77].

The children who took part in the study were primary school children, an age range that has recently seen increasing use in HRI studies [4, 27, 50, 60], as means of supplementing existing educational practice [29]. In terms of generalising the results to other children of the same age, the UK government Office for Standards in Education, Children’s Services and Skills (Ofsted) conducts regular school inspections and compiles national statistics and performance tables [78]. For the school at which this study was conducted, the proportion of children who attained the expected standard in reading, writing and mathematics (72%, 2014 rating) for the age group (Key Stage 2, level 4) is consistent with the regional (74%) and national (78%) mean ratings. Based on this characterisation, we suggest that the results could be reasonably generalised to other primary school populations (at least in the U.K.), thus supporting the wider applicability of the findings.

One further point of note is the wider effect of the presence of the robots in the classroom. The teacher debriefing highlighted the impact of novelty: in the first week of the experiment, some disruption to the class occurred as children were distracted by the robot actions and speech. However, they noted that in both classrooms, this distracting effect dissipated in the second week, although they reported still being able to use the robots as a motivator for the children [79]. This is supported by the high levels of interest/enjoyment in the activity at the end of the study (non-significantly higher for the personalised condition). This maintenance of motivation speaks to the wider role of technology, including social robotics, in the classroom and how it is handled (‘orchestrated’) by the teachers [80]. While acceptance was high in the present study, this may be a self-selection bias (i.e. the school and teachers were enthusiastic about the study prior to implementation), and further examination of the effort required on the part of the teachers and the school versus the learning benefits afforded by the type of personalised social robot systems we have demonstrated here is necessary, particularly in embedded applications (i.e. inside the classroom itself), as we have achieved in the present study.

The methodology employed, with the autonomous robots embedded (both physically and in terms of curriculum) within primary school classes without experimenter supervision, maximises the ecological validity of the study, and thus the implications for educational practice and application. This study found that a robot peer exhibiting personalised behaviours in a collaborative learning task with individual children facilitated improved learning for the children in a novel task over a non-personalised robot behaviour. This effect was not seen for the familiar task, and whilst a differential improvement was observed in the incidental task, these results require further verification in light of the non-significant differences between the child perceptions. We conclude that while further empirical study is required to distinguish between, and indeed maximise the impact of, the different aspects of personalisation employed, we have shown that robot personalisation provides a positive influence on child learning in the classroom.

## Supporting information

S1 File Robot behaviour details and questionnaires. Full details of the robot behaviour in the two experimental conditions, and transcript of the questionnaires used in the post-study debriefing.
(PDF)

S2 File Experimental data spreadsheet. All details of the experimental data, organised by spreadsheet tab, including pre/post and within-interaction learning and performance data, and screen question and questionnaire responses.
(ZIP)
