# Supplementary material for: Robot education peers in a situated primary school study: Personalisation promotes child learning
Source: PLoS One. 2017 May 23;12(5):e0178126. doi: 10.1371/journal.pone.0178126 (PMC5441605; doi:10.1371/journal.pone.0178126)
Supplement: S1 File — Full details of the robot behaviour in the two experimental conditions, and transcript of the questionnaires used in the post-study debriefing. (PDF) [file pone.0178126.s001.pdf]

Robot Education Peers in a Situated Primary School Study: Personalisation Promotes Child Learning

Supplementary Materials

– S1: Robot behaviour details and questionnaires –

Paul Baxter*, Emily Ashurst, Robin Read, James Kennedy, Tony Belpaeme
pbaxter@lincoln.ac.uk

Robot Behaviours

Verbal Behaviour

Example phrases used by the robots in the two conditions are shown in table S1-1. The task related information (e.g. feedback) and number of utterances were matched in both conditions; only the style of delivery varied between conditions. In the Personalised condition an informal style was applied, with the occasional use of the interacting child's name. In the Non-Personalised condition an imperative style was used, with no use of the interacting child's name. For the feedback during the interaction (both good and bad move feedback) a set of alternative phrases, in the style of the condition, was defined. During the interaction a random selection was made each time feedback was required.

Table S1-1: Verbal Phrases Used by the Robot. Examples of the phrases used in the two conditions for a number of events in the interaction. For a number of these, a set of phrases was pre-defined and randomly chosen from when the relevant event occurred. An arbitration mechanism was put in place to ensure that phrases did not overlap (particularly applying to the verbal move feedback).

Interaction Stage	Personalised Condition (P)	Non-Personalised Condition (NP)
Greeting	"Hello! Find your name on the screen and we can continue"	"Find your name on the screen and we will start"
Interaction start	"Welcome back *name*! You've been doing well so far, let's continue!"	"Hello, we will do a sorting task now."
Good move feedback	"good job"	"right answer"
Bad move feedback	"better luck next time"	"that is not correct"
Stone age factoid	"Did you know that the stone-age was two million years ago?"	"The stone-age was two million years ago"
Interaction end	"Time to go back to class! But can you answer the quick question on the screen first please?"	"Time to stop! Answer the quick question on the screen, and then go back to class!"
Answer acknowledgement	"Thank you for that! Time for me to rest!"	"Answer recorded. My rest period will now start."

Non-verbal Behaviour

The non-verbal behaviour of the robot that differed between the conditions P and NP is shown in table S1-2. In both the conditions, the robot would be crouched when not engaged in an interaction with a child, and would stand when an interaction took place. Also in both conditions, the robot would make touchscreen-oriented movements: a simulation of the robot moving items displayed on the touchscreen, where the robot arm movement is synchronised with the movement of the item on the screen. The robot head would also reorient to follow the movement on the screen.

Table S1-2: Non-Verbal Robot Behaviours. Outline of non-verbal behaviours used in the P and NP conditions.

Aspect of Behaviour	Personalised Condition (P)	Non-Personalised Condition (NP)
Gaze	Socially responsive gaze: robot head direction oriented in direction of child's face when oriented towards the robot	Robot would maintain a consistent gaze direction throughout the interaction
Move adaptation	Alignment of touchscreen-oriented robot behaviours: speed, delay and accuracy of categorisation moves	Robot behaviour did not change over time, or in response to child behaviour
Anticipatory reaction	Robot would stand in preparation for an interaction if a child was observed to approach	Robot would remain crouched until a child pressed the start button on the touchscreen
Idle behaviour	Life-like posture movements of head and arms, in addition to touchscreen-oriented movements	Static postures; the only movements are touchscreen oriented

Questionnaires

The full set of questions administered before and after the end of the experimental period to the children is detailed below. The motivation and references related to prior use in the literature and validation may be found in the main paper (Methods section).

Pre-Experiment Expectation Questions

These four questions were administered before the experiment had taken place, and at the same time as the pre-experiment knowledge test.

(a) For me, I think playing with the robot will be like playing with:
- a toy
- a games console
- a friend
- a pet
- other

(b) Do you usually play outdoors or indoors?
- outdoors
- indoors

(c) Do you want to know how the robot works?
1– not at all
2– not really
3– a bit
4– yes
5– yes, very much

(d) Do you want to be friends with the robot? *(reversed scoring)*
1– yes, very much
2– yes
3– a bit
4– not really
5– not at all

Social Presence Questionnaire

This questionnaire was administered after the end of the experimental period in a paper-based format. For each of the following questions, the children were asked to select one option from the following: (1) Not at all; (2) Not really; (3) A bit; (4) Yes; (5) Yes very much.

(a) I noticed the robot.

(b) The robot noticed me.

(c) I kept paying attention to the robot.

(d) The robot kept paying attention to me.

(e) I knew what the robot was thinking.

(f) The robot knew what I was thinking.

(g) I could tell how the robot was feeling.

(h) The robot could tell how I was feeling.

(i) I was influenced by the robot's moods.

(j) The robot was influenced by my moods.

(k) I followed what the robot did.

(l) The robot followed what I did.

Social Support Questionnaire

This questionnaire was administered after the end of the experimental period in a paper-based format. For each of the following questions, the children were asked to select one option from the following: (1) Not at all; (2) Not really; (3) A bit; (4) Yes; (5) Yes very much.

(a) The robot comforts me when I am feeling upset.

(b) The robot cares about me.

(c) The robot gives me good advice.

(d) The robot accepts me for who I am.

(e) The robot supports my decisions.

(f) I can count on the robot.

(g) The robot encourages me.

(h) The robot understands me.

(i) The robot praises me when I have done something well.

Interest/Enjoyment Questionnaire

This questionnaire was administered after the end of the experimental period in a paper-based format. For each of the following questions, the children were asked to select one option from the following: (1) Not at all; (2) Not really; (3) A bit; (4) Yes; (5) Yes very much. Items with reverse scoring are indicated.

(a) I enjoyed doing this activity very much.

(b) This activity was fun to do.

(c) I thought this was a boring activity. *(reversed scoring)*

(d) This activity did not hold my attention at all. *(reversed scoring)*

(e) I would describe this activity as very interesting.

(f) I thought this activity was quite enjoyable.

(g) While I was doing this activity, I was thinking about how much I enjoyed it.

Perceived Competence Questionnaire

This questionnaire was administered after the end of the experimental period in a paper-based format. For each of the following questions, the children were asked to select one option from the following: (1) Not at all; (2) Not really; (3) A bit; (4) Yes; (5) Yes very much.

(a) I think I did pretty well at this activity, compared to other classmates.

(b) I was pretty skilled at this activity.

Additional Data

Equivalent Exposure to Learning Material in Both Conditions

Between the two conditions, there was a difference in the number of interactions with the robot ($M_P = 3.867$, $n_P = 30$, 95% CI=[3.743,3.990], $M_{NP} = 2.929$, $n_{NP} = 29$, 95% CI=[2.768,3.089], independent samples two-tailed t-test: $t(57)$=9.109, p <.0001). However, children in the two conditions had a similar level of exposure to the material to be learned (both novel and familiar): the total number of completed libraries is not significantly different between the two conditions ($M_P = 13.167$, $n_P = 30$, 95% CI=[12.203,14.130], $M_{NP} = 14.241$, $n_{NP} = 29$, 95% CI=[12.505,15.978], independent samples two-tailed t-test: $t(57)$=1.070, p=0.289). Taken together, these metrics indicate that in condition P the children went through the material with the robot at a slower rate than children in the NP condition. Despite the difference in number of interactions, this equivalence in exposure to the learning material means that it is justified to consider learning effects.

Image Library Progression Significances

For all image libraries, performance in the Personalised condition exceeds that in the Non-Personalised condition, however, significance is only present in a few of these cases (table S1-3).

Table S1-3: Comparing performance in each of the image libraries. Sample mean, 95% confidence intervals (CI), and sample size are shown per condition. Test statistic used is an unpaired two-tailed t-test, unequal variance. *P-values highlighted in red are significant at the .05 level.*

Lib	Condition P	Condition NP	Significance
1	$M_{p1} = 0.936$, CI=[0.902,0.970], n=30	$M_{np1} = 0.877$, CI=[0.782,0.971], n=29	t(35)=1.152, p=.257
2	$M_{p2} = 0.798$, CI=[0.761,0.836], n=30	$M_{np2} = 0.738$, CI=[0.654,0.823], n=29	t(39)=1.275, p=.209
3	$M_{p3} = 0.937$, CI=[0.901,0.973], n=30	$M_{np3} = 0.886$, CI=[0.844,0.928], n=29	t(55)=1.802, p=.076
4	$M_{p4} = 0.765$, CI=[0.728,0.802], n=30	$M_{np4} = 0.682$, CI=[0.620,0.743], n=28	t(44)=2.248, p=.029
5	$M_{p5} = 0.791$, CI=[0.732,0.850], n=30	$M_{np5} = 0.739$, CI=[0.674,0.805], n=28	t(55)=1.15, p=.254
6	$M_{p6} = 0.813$, CI=[0.784,0.842], n=30	$M_{np6} = 0.779$, CI=[0.717,0.841], n=28	t(38)=0.959, p=.343
7	$M_{p7} = 0.720$, CI=[0.644,0.795], n=30	$M_{np7} = 0.717$, CI=[0.639,0.794], n=28	t(56)=0.054, p=.957
8	$M_{p8} = 0.749$, CI=[0.708,0.791], n=29	$M_{np8} = 0.637$, CI=[0.560,0.714], n=28	t(40)=2.483, p=.017
9	$M_{p9} = 0.763$, CI=[0.697,0.829], n=28	$M_{np9} = 0.753$, CI=[0.674,0.832], n=26	t(49)=1.371, p=.904
10	$M_{p10} = 0.845$, CI=[0.805,0.885], n=29	$M_{np10} = 0.710$, CI=[0.629,0.790], n=25	t(35)=2.919, p=.006
11	$M_{p11} = 0.674$, CI=[0.589,0.760], n=27	$M_{np11} = 0.568$, CI=[0.486,0.650], n=21	t(45)=1.742, p=.088
12	$M_{p12} = 0.780$, CI=[0.718,0.842], n=16	$M_{np12} = 0.617$, CI=[0.504,0.729], n=17	t(24)=2.434, p=.022
13	$M_{p13} = 0.771$, CI=[0.703,0.838], n=12	$M_{np13} = 0.750$, CI=[0.694,0.806], n=15	t(23)=0.475, p=.639
14	$M_{p14} = 0.680$, CI=[0.597,0.763], n=12	$M_{np14} = 0.473$, CI=[0.341,0.604], n=13	t(20)=2.62, p=.016
15	$M_{p15} = 0.656$, CI=[0.468,0.844], n=8	$M_{np15} = 0.507$, CI=[0.464,0.551], n=11	t(8)=1.513, p=.169
16	$M_{p16} = 0.851$, CI=[0.624,1.079], n=5	$M_{np16} = 0.590$, CI=[0.494,0.686], n=9	t(5)=2.073, p=.088
17	$M_{p17} = 0.857$, CI=[0.577,1.137], n=2	$M_{np17} = 0.438$, CI=[0.264,0.611], n=5	t(2)=2.496, p=.140
18	$M_{p18} = 0.697$, CI=[0.638,0.756], n=2	$M_{np18} = 0.686$, CI=[0.555,0.818], n=4	t(4)=0.144, p=.892
